# Spine SBRT With Halcyon™: Plan Quality, Modulation Complexity, Delivery Accuracy, and Speed

**DOI:** 10.3389/fonc.2019.00319

**Published:** 2019-04-26

**Authors:** Heather M. Petroccia, Irina Malajovich, Andrew R. Barsky, Alireza Fotouhi Ghiam, Joshua Jones, Chunhao Wang, Wei Zou, Boon-Keng Kevin Teo, Lei Dong, James M. Metz, Taoran Li

**Affiliations:** ^1^Department of Radiation Oncology, Perelman Center for Advanced Medicine, University of Pennsylvania, Philadelphia, PA, United States; ^2^Department of Radiation Oncology, Duke University Medical Center, Durham, NC, United States

**Keywords:** multi-leaf collimator, SBRT, spinal metastasis, Halcyon™, plan quality, modulation complexity score

## Abstract

**Purpose:** Spine SBRT requires treatment plans with steep dose gradients and tight limits to the cord maximal dose. A new dual-layer staggered 1-cm MLC in Halcyon™ treatment platform has improved leakage, speed, and DLG compared to 120-Millennium (0.5-cm) and High-Definition (0.25-cm) MLCs in the TrueBeam platform. Halcyon™ 2.0 with SX2 MLC modulates fluence with the upper and lower MLCs, while in Halcyon™ 1.0 with SX1 only the lower MLC modulates the fluence and the upper MLC functions as a back-up jaw. We investigated the effects of four MLC designs on plan quality for spine SBRT treatments.

**Methods:** 15 patients previously treated at our institution were re-planned according to the NRG-BR-002 guidelines with a prescription of 3,000 cGy in 3 fractions, 6xFFF, 800 MU/min, and 3-arc VMAT technique. Planning objectives were adjusted manually by an experienced planner to generate optimal plans and kept the same for different MLCs within the same platform.

**Results:** All treatment plans were able to achieve adequate target coverage while meeting NRG-BR002 dosimetric constraints. Planning parameters were evaluated including: conformity index, homogeneity index, gradient measure, and global point dose maximum. Delivery accuracy, modulation complexity, and delivery time were also analyzed for all MLCs.

**Conclusion:** The Halcyon™ dual-layer MLC can generate comparable and clinically equivalent spine SBRT plans to TrueBeam plans with less rapid dose fall-off and lower conformity. MLC width leaf can impact maximum dose to organs at risk and plan quality, but does not cause limitations in achieving acceptable plans for spine SBRT treatments.

## Introduction

Stereotactic body radiotherapy treatment (SBRT) for metastatic spinal tumors necessitates radiation treatment plans with high dose targets immediately adjacent to the spinal cord. To achieve uniform coverage while maintaining safe doses to the spinal cord, steep dose gradients must be achieved with precise dose delivery. Due to the high stakes of delivering high dose which can cause myelitis in the spinal cord, spine SBRT delivery requires dosimetric accuracy and robust patient immobilization/positioning (1, 2). Accurate plan delivery, including dose rate modulation, gantry position, collimator position, and multi-leaf collimator (MLC) position, are required to ensure sharp dose fall-off.

A new dual-layer staggered 1-cm wide MLC in Halcyon™ treatment platform (Varian Medical System, Palo Alto, CA) has reduced leakage, increased speed, and improved dosimetric leaf gap (DLG), as compared to Millennium-120 MLC with 0.5 cm and High-Definition-120 (HD) MLC with 0.25 cm centrally located leaf widths associated with the TrueBeam platform (Varian Medical System, Palo Alto, CA). Despite the larger 1.0-cm leaf width, Halcyon™ 2.0 with SX2 MLC has the ability to modulate fluence with both the proximal and distal MLC banks, while Halcyon™ 1.0 with SX1 only the distal MLCs modulates the fluence and the proximal MLCs functions as a backup jaw by moving to the most distally extended lower leaf pair. While the leaf size for the new dual-layer staggered 1-cm MLC is larger than the leaf size of the Millennium (0.5 cm) and HD MLC (0.25 cm), the Halcyon™ platform has two times faster leaf speed (5 cm/s), four times faster collimator rotation (2.5 RPM), and four times faster gantry speed (4 RPM) as compared to the TrueBeam (TB) platform. The dual-layer MLC has 77 mm leaf thickness and has low leaf transmission; therefore, no backup jaw is necessary.

In this study, we investigate the ability of dual-layer staggered 1-cm MLC, in both Halcyon™ 1.0 and 2.0, to generate treatment plans with conformal dose distributions for complex target volumes with steep dose gradients as compared to treatment plans generated with the Millennium and HD MLC by analyzing the following planning parameters: conformity index (CI), homogeneity index (HI), gradient measure (index), and global point dose maximum. Since the diameter of the spinal cord is comparable in size to the 1-cm MLC leaf width, this study evaluates the ability of the dual-layer staggered 1-cm MLCs to modulate fluence using an effective leaf width of 0.5 cm by examining the percentage of shaping performed by distal and proximal MLCs, the modulation complexity score, the total MU, and the gamma analysis passing rate metric for delivered plans. The speed of beam delivery is also compared between all four modalities.

Previous studies have analyzed the effects of MLC leaf width on plan quality for stereotactic body radiation therapy (1, 3–5). While dosimetric differences are generally small, differences in conformality, heterogeneity, and a decrease in the volume of the normal tissue being irradiated have been reported by varying MLC leaf width size (4, 5). In general, smaller MLC leaf widths have been shown to generate either similar or improved plan quality when compared to larger MLC leaf widths for SBRT. In this study, we investigate how the 1-cm dual layer MLC influences plan quality compared to conventional single layer MLC treatment methods and determine if Halcyon™ generates clinically acceptable plans with equivalent quality for spine SBRT.

## Materials and Methods

### Study Cohort

Fifteen patients with metastatic spinal tumors treated with spine SBRT in the *de novo* or post-operative setting at the University of Pennsylvania between November 2016 and January 2018 were retrospectively identified under Institutional Review Board approval (#829182). For the study, all patients were re-planned with a total prescription dose of 3,000 cGy delivered in 3 fractions according to the standard dose fractionation used in NRG protocol BR-002 (6).

**Figure 1:**
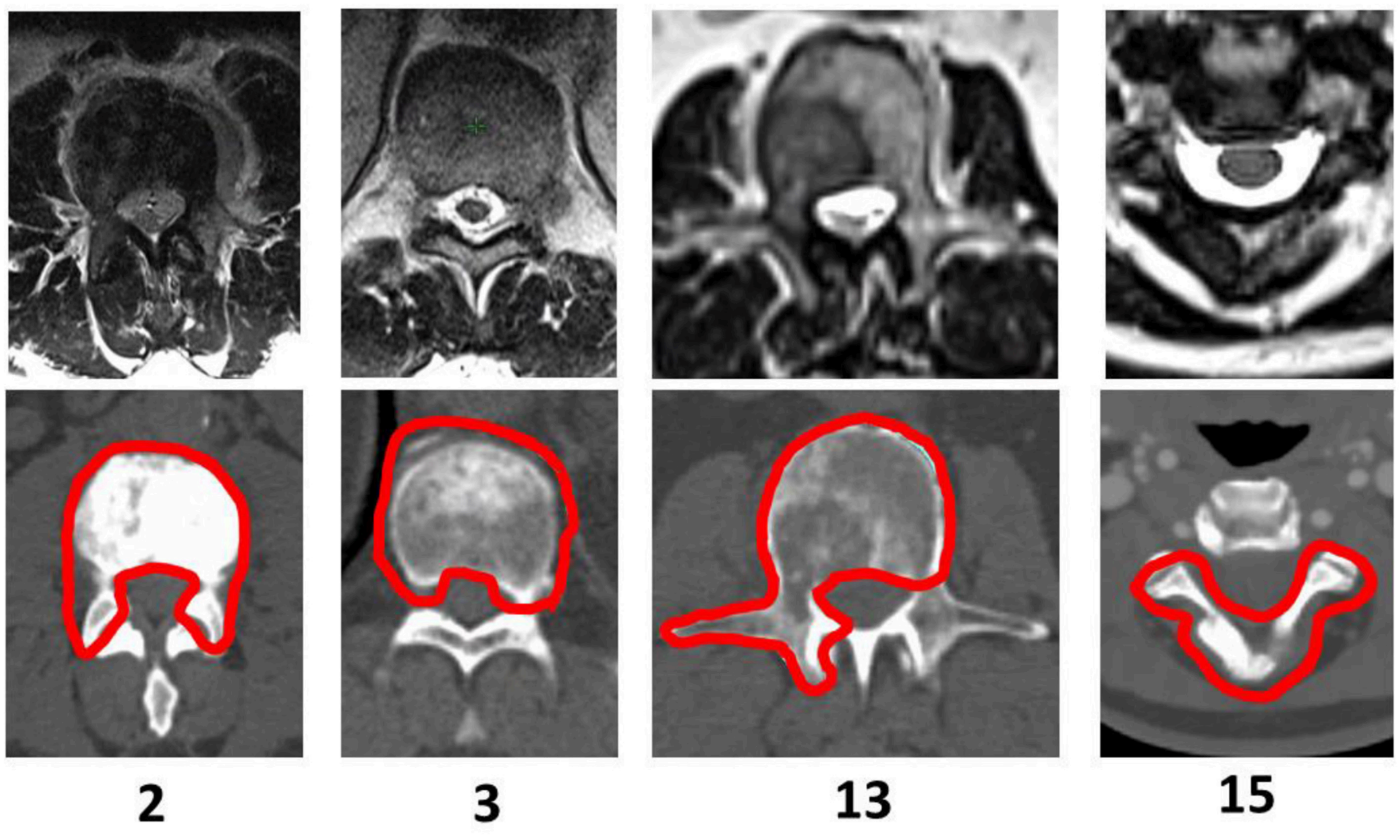
PTV Shapes—Examples of selected patients treated with spine SBRT. The CT slice displayed corresponds to the location of the cord dose maximum for Halcyon™ 1.0 generated treatment plans.

Clinical target volumes (CTV) were delineated by attending physicians in accordance with the International Spine Research Consortium recommendations (7, 8). Expansion from CTV to planning target volume (PTV) includes a 0 to 2 mm margin, which was cropped away from the thecal sac to prevent overlap between structures. Examples of PTV volumes are shown in Figure 1. The true cord was delineated using an axial T2-weighted MR scan which was obtained on the same day as the CT scan acquired for simulation. The CT scans were acquired with slice thicknesses varying between 2 and 3 mm. Spinal cord volumes extend ~5–6 mm superior and inferior to the target volume.

A description of PTV location, geometry, and complexity as indicated by the classification by Cox et al. (7) for the 15 cases selected for this study and Bilsky scores (9) is displayed in Table 1. For 12 of the cases, only a single vertebral body was treated, and for 3 cases more than 1 vertebral body was treated.

**Table 1:** Study Population−15 patients were retrospectively selected under IRB approval, who were treated for spinal metastasis using a stereotactic body radiotherapy approach. The tumor location, length of PTV, the number of vertebral bodies treated, de novo or post-operative setting, and corresponding Bilsky scores are reported below.

**Case #**	**# of vertebral bodies**	***De novo*/post-operative**	**Cervical/thoracic/lumbar**	**Length of PTV (cm)**	**Classification by Cox et al. (7)**	**Bilsky score**
1	1	*De novo*	Thoracic–T9	2.7	1,2,6	1b
2	1	*De novo*	Lumbar–L3	3.2	1,2,3,5,6	N/A
3	1	*De novo*	Thoracic–T11	2.8	1	0
4	1	*De novo*	Thoracic–T10	3.0	1,2,5,6	1b
5	1	*De novo*	Thoracic–T3	2.7	1	0
6	1	*De novo*	Lumbar–L3	3.6	1,2,3	N/A
7	1	*De novo*	Thoracic–T5	2.0	1,2,3,4,5,6	1a
8	1	Post-op	Thoracic–T4	2.6	1,2,3,5,6	1c
9	1	*De novo*	Thoracic–T6	3.0	1,2,6	0
10	1	*De novo*	Thoracic–T10	1.8	1,2,6	1a
11	1	*De novo*	Thoracic–T10	3.0	1,2,6	0
12	3	Post-op	Thoracic–T3 to T5	4.5	1,2,5,6	2^*^
13	2	*De novo*	Lumbar–L2 to L3	6.8	1,2,5,6	0
14	3	Post-op	Thoracic & Cervical–C6 to T1	7.2	1,2,3	1c^*^
15	1	*De novo*	Cervical–C2	2.7	3,4,5	0

* pre-op Bilsky score

### Treatment Plan Parameters

All plans are generated within Eclipse™ treatment planning software (v15) using a 6X flattening filter free (FFF) energy and 800 MU/min dose rate to minimize differences between treatment units and directly compare MLCs. An evaluation of plan quality varying the total number of arcs between 2 and 5 was performed for Halcyon™ plans. Based on the results of this study, all plans use a 3-arc VMAT approach for optimization and delivery. Collimator angles are selected to be 10°, 350°, and 30°/45° for TrueBeam plans according to our institutional guidelines. The optimal collimator angle for the Halcyon™ plans is determined automatically by the treatment planning system (TPS), thus resulting in all plans having the same collimator angles of 285°, 345°, and 45° despite differences in target geometry. This method mimicked actual clinical scenario of determining collimator rotations for both platforms. For each case, planning objectives are adjusted manually by an experienced planner to generate optimal plans for each platform and kept the same for different MLCs within each respective platform. Once acceptable plan objectives are determined, the objectives are entered prior to commencing optimization and no further adjustments are made.

### Dosimetric Evaluation Parameters

Planning parameters are compared between the Halcyon™ and TrueBeam platforms to evaluate plan quality. All plans are optimized using Photon Optimizer (PO) algorithm (Version 15.1), where the structures, DVH calculation, and dose sampling are defined spatially with a single matrix (10), and calculated with 0.1 cm grid. Jaw tracking is turned on for TrueBeam plans. All plans are normalized so that at least 90% of the prescription dose covers 100% of the target volume. In this study, the homogeneity index (HI) is defined as D98%/D2% of the PTV; the conformity index (CI) is defined as the treated volume enclosed by the 100% isodose line as compared to the PTV (11); the gradient measure is defined by the TPS as the difference between the equivalent sphere radius of the prescription and 50%-prescription doses (10); and the global maximum is the dose calculation to 0.1 cc.

To determine statistical significance, matched pair comparisons were performed comparing the difference between SX1, SX2, Millennium MLCs compared to the HD MLC, and the results are shown in **Figure 3B**. Two-tailed paired *t*-test was used to determine statistical significance with threshold of *p* = 0.05.

### Evaluation of Delivery Accuracy, Modulation Complexity, and Speed

Delivery accuracy, modulation complexity, and speed of delivery are investigated for all four MLC types. Measurements are acquired for all plans with the ArcCheck (SunNuclear, Melborne, Florida) in absolute dose mode and analyzed using a gamma index metric with a criterion of 2%/2 mm with a 90% passing rate and a 10% low dose threshold. Next, we investigated how much additional modulation is provided by the proximal MLC between SX2 and SX1 by analyzing the amount of leaf shaping given for leading and trailing leaves. The control points are super-sampled for all plans, and the distal and proximal leaf positions are compared to determine furthest extended leaf edge. Further investigation in the amount of modulation between MLCs is performed by calculating the modulation complexity score (MCS) for each case (12). For speed analysis, prior to plan delivery all TrueBeam plans are adjusted to be 1,400 MU/min as compared to the 800 MU/min in the Halcyon™ platform to utilize optimal delivery characteristics for each treatment unit.

## Results

### Spinal Cord Point Dose Maximum

**Figure 2:**
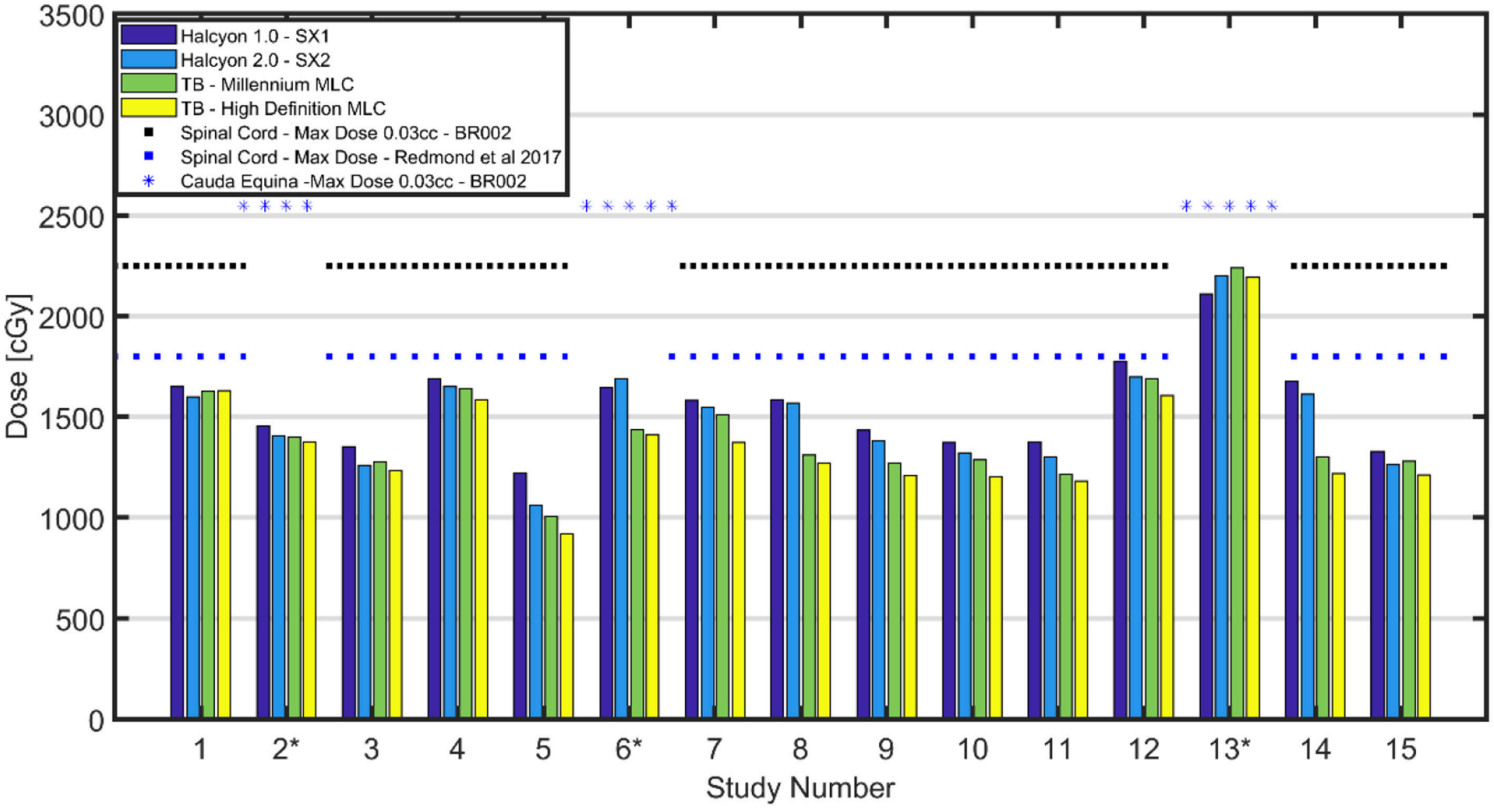
Cord Max Dose Comparison**—**Comparison of cord max dose or cauda equina (D0.03 cc) across all patients. Black dotted lines shown above correspond to the OAR dose limits for 3 fraction SBRT as defined in the NRG-BR002 protocol for spinal cord for a volume <0.03 cc. A spinal cord constraint for 3 fractions of 18-21 Gy D_max_ displayed as a blue dotted line (8). All plans have cord or cauda equina under the limit set in NRG-BR002. Maximum dose to spinal cord was found to have a range of [1,060–1,698] cGy for the Halcyon™ 2.0 with SX2, while the TrueBeams with Millennium MLC and HD MLC were found to have comparable maximum doses ranges of [1,006–1,688] cGy and [919–1,631] cGy, respectively.

The maximum dose to 0.03 cc for the spinal cord and cauda equina are reported in Figure 2. A dotted black line shown in Figure 2 is NRG-BR002 recommendation limiting volume of <0.03 cc to a maximum dose of 22.5 Gy and a volume of <1.2 cc to a dose of 13 Gy for the spinal cord for a 3 fraction SBRT treatment. A blue dashed line in Figure 2 indicates recommendation by Redmond et al. (8) limiting the maximum point dose to the cord to 18–21 Gy for 3 fraction treatment (no prior RT and no cord compromise). All plans meet the spinal cord constraint by Redmond et al. (8). In a matched pair study, the difference between 0.03 cc cord dose maximum for SX2 and SX1 plans and between HD MLC and SX2 plans is found be statistically significant (*p* < 0.02), with a mean difference of 52.1 (28.6,75.8) cGy and 110.8 (46.8,174.7) cGy lower, respectively. No statistically significant difference in 0.03 cc cord dose maximum between Millennium MLC and SX2 plans is found.

Furthermore, for all cases except for the 2 post-operative cases, 12 and 14, the volumetric constraint of <1.2 cc receiving a total dose of 13 Gy is met, and no statistically significant (*p* < 0.05) difference in dose is found between the different MLCs. Two patients, who failed to meet the volumetric constraint, had metal hardware within the PTV, and their target included 3 adjacent vertebral bodies. As marked with an asterisk in Figure 2, cases 2, 6, and 13 are evaluated for maximum dose to the cauda equina (surrogate of the cord). NRG-BR002 recommends a volume of <0.03 cc of the cauda equina to receive <25.5 Gy, and all cases meet this dosimetric constraint.

### Dosimetric Evaluation Results

**Figure 3:**
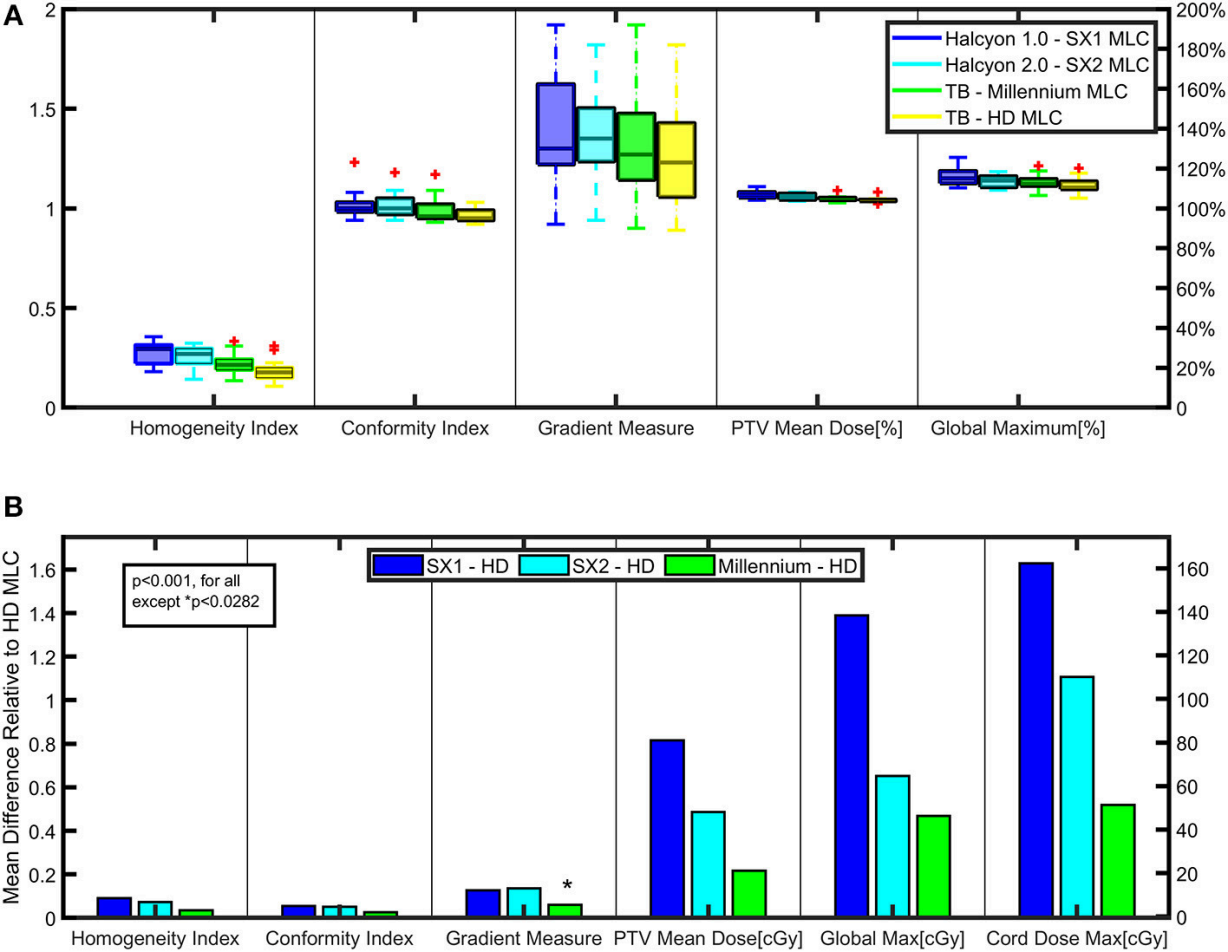
Key Dosimetric Parameters—**(A)** Planning parameters are compared between the Halcyon™ and TrueBeam platforms to evaluate plan quality. **(B)** Matched paired analysis was performed comparing the difference between SX1, SX2, and Millennium-120 MLC to the High Definition-120 (HD) MLC for various plan parameters to evaluate statistically significant trends.

Plan parameters are displayed in Figure 3A, including the HI, CI, gradient measure, PTV mean dose, and the global dose maximum (0.1 cc) for all plans. Halcyon™ SX1 and SX2 plans are shown to have similar conformity and homogeneity (SX2: CI 1.0±0.06, HI 0.27±0.05; SX1: CI 1.0±0.07, HI 0.29±0.05) as compared to the TrueBeam platform (HD: CI 0.95±0.03, HI 0.18±0.06; Millennium: CI 0.96±0.07, HI 0.21±0.06); however, the gradient measure, as defined by Eclipse, indicates the TrueBeam platform has steeper dose fall-off than the Halcyon™ platform. The gradient index, as defined by the ratio of the 50% prescription isodose volume to the volume of the prescription isodose, are 4.1±0.7, 4.2±0.7, 3.8±0.7, and 3.6±0.5, respectively for SX1, SX2, Millennium, and HD MLC (13). A trend of improved dose fall-off is indicated between the Halcyon™ and TrueBeam platform. Plans generated within the Halcyon™ platform have higher maximum dose than plans generated using the TrueBeam platform. Global Dmax of plans generated using SX1 and SX2 are 119.1±3.4% and 121.2±4.6% (normalized to Rx) which are higher than HD MLC plans with global Dmax of 115.7±1.3%.

### IMRT QA Results

Delivery accuracy for the 4 different treatment modalities are measured and analyzed using 2%/2 mm gamma index passing metric. All plans for all modalities met the criteria of above 90% pass rate. The passing rates for Halcyon™ 1.0 and version 2.0 are 98.8 ± 0.2% and 96.9 ± 2.0%, respectively. No statistically significant difference in gamma analysis passing rate is observed across four types of MLCs.

### Comparison of Delivery Accuracy and Efficiency for All Modalities

**Figure 4:**
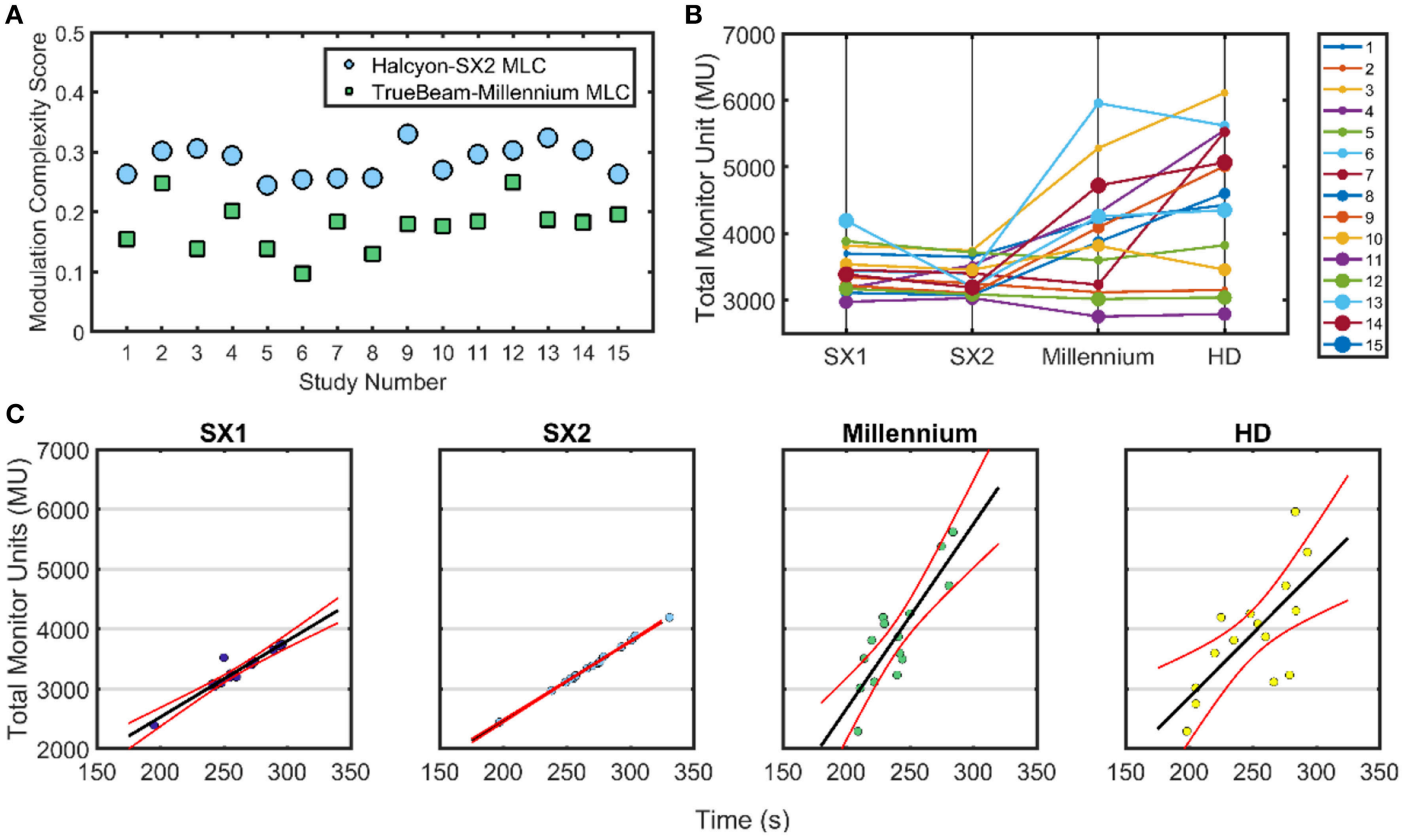
Treatment Delivery Parameters**—**Delivery parameter including total MU, modulation complexity score, and delivery time is compared for the Halcyon™ platform with SX1 and SX2 and TrueBeam platform with High Definition (HD) 120 MLC and Millennium 120 MLC. **(A)** The modulation complexity score is shown for Halcyon™ version 2 with SX2 is compared to TrueBeam Millennium MLC. **(B)** Shows the total MU delivered for each of the different treatment modalities. **(C)** All TrueBeam plans were adjusted to be 1,400 MU/min as compared to the 800 MU/min available in the Halcyon™ platform to utilize optimal delivery characteristics per treatment unit. Linear fits with the 95% confidence interval for delivery time compared to the total MU for all patients with the exception of case 3 due to exceptionally high MU.

Figure 4B displays a comparison of the combined total MU for the 3-arc VMAT delivery that is analyzed for all modalities. 6 of 15 cases show an increase in MU between Halcyon™ and TrueBeam treatment platforms with a general trend of plans generated using the HD MLC having the largest MU. In Figure 4C, linear fits with the 95% confidence interval are shown for delivery time vs. MU for all four different MLCs. Paired analysis showed no statistical difference between TrueBeam HD MLC and Halcyon™ platform, and small (avg. 20–30 s) but significant (*p* < 0.02) difference between TrueBeam Millennium MLC and Halcyon™ MLCs. The plan generated for the Halcyon™ 2.0 with SX2 delivery time is shown to be 4.32 ± 0.42 min compared to the delivery time of 3.99 ± 0.40 min for the TrueBeam with Millennium MLC.

### Modulation Complexity Evaluation

#### Halcyon™ 1.0 Compared With Halcyon™ 2.0

Halcyon™ 2.0 plans showed no statistical difference compared to Halcyon™ 1.0 plans in quality for homogeneity and conformity. Although Halcyon™ 1.0 appears to have steeper dose fall-off than Halcyon™ 2.0 as indicated by the gradient measure in Figure 3A, a matched paired analysis showed no statistical significance (p>0.05). Halcyon™ 1.0 plans are consistently hotter on average than Halcyon™ 2.0 by 2%. Investigating the leaf position of the leading and trailing leaves of both the proximal and distal MLC banks for each control point, the proximal MLC is found to help shape the field by extending past the distal leaf edge on average by 29.5 ± 6.2% of the time in Halcyon™ 2.0.

#### Halcyon 2.0 Compared With Millennium-120 MLC

Since Halcyon™ 2.0 plans modulated fluence with both the distal and proximal MLCs, the effective leaf width has the potential to be 0.5 cm, which is the same leaf size as the Millennium MLC. Thus, if the Halcyon™ 2.0 achieves the effective 0.5 cm leaf width, plan quality should be similar to the TB with Millennium MLC. Halcyon™ 2.0 plans have no statistically significant difference in mean dose to PTV and global dose maximums compared to plans generated with the Millennium MLC; however, the 50% dose fall-off is much higher in Halcyon™ 2.0 as compared to Millennium MLC. Further analysis is performed to investigate why the gradient index for Halcyon™ 2.0 is not closer in value to the Millennium MLC. The MCS is evaluated for both modalities as shown in Figure 4A. A lower MCS indicates higher modulation. The TrueBeam Millennium MLC is shown to result in substantially higher modulation with an average MCS of 0.18 ± 0.04 for all plans as indicated by the lower value for all cases compared to the Halcyon™ 2.0 plans with an average MCS of 0.28 ± 0.03.

## Discussion

### Comparison of TrueBeam to Halcyon™ Platform

**Figure 5:**
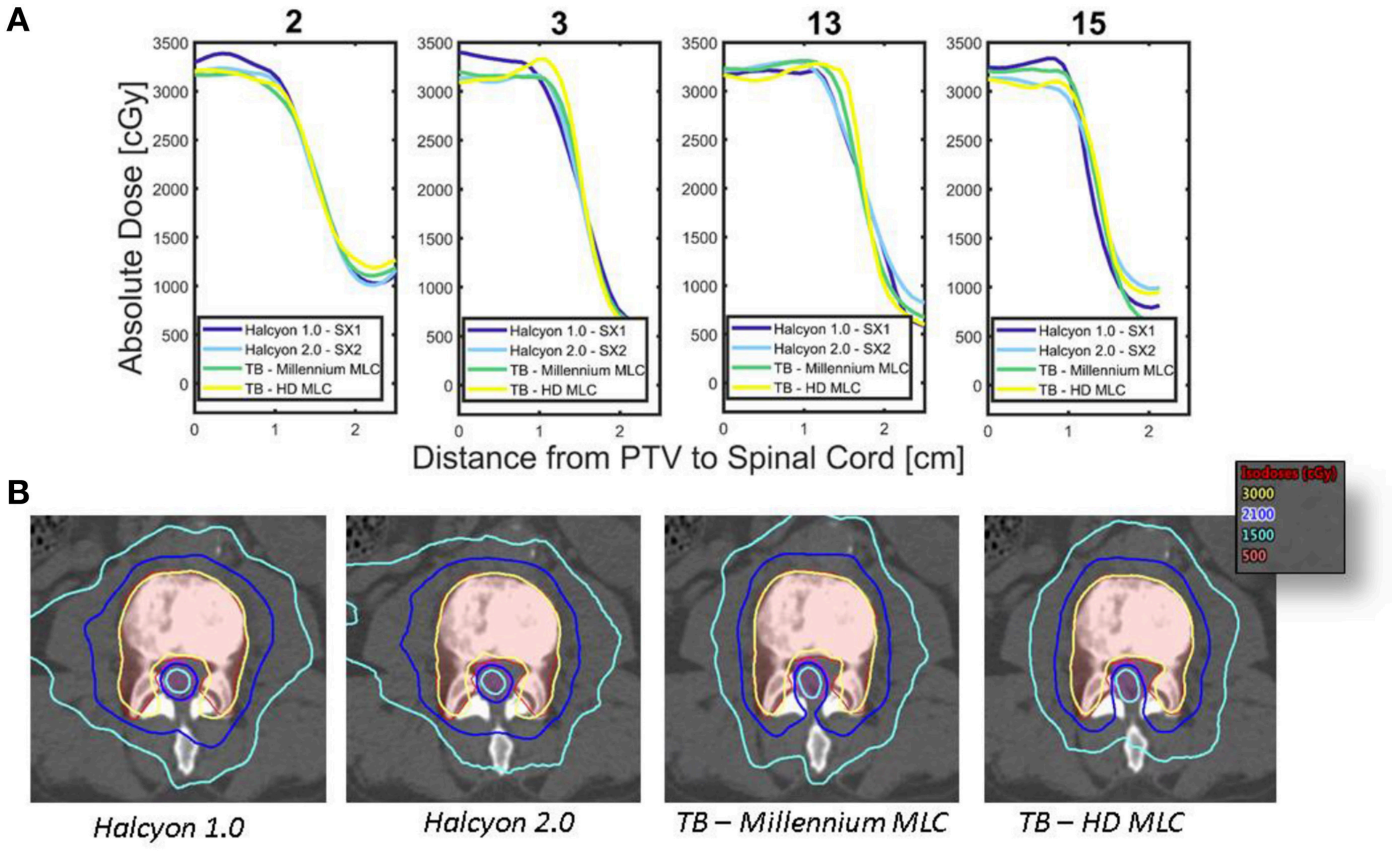
Dose Fall-off inside Spinal Canal—**(A)** Dose profiles originating 1 cm from the edge of the PTV moving posteriorly toward the spinal cord are shown for each of the different treatment modalities for 4 patients. **(B)** Comparison of the isodose lines between various treatment platforms for case 2 are shown, thus displaying the conformality of the dose to the target volume as well as the fall off toward the spinal cord. PTV is shown as red translucent contour and likewise the spinal cord is shown as the magenta translucent contour.

In this study we evaluated the Halcyon™ platform's performance for spine SBRT. Halcyon™ treatment plans generated with SX1 and SX2 are shown to have a similar CI and HI as compared to the TrueBeam platform; however, the gradient measure indicates that TrueBeam plans have steeper dose fall-off than the Halcyon™ platform and an increase in modulation as indicated by MCS for the TrueBeam Millennium MLC as compared to the Halcyon™ 2.0 plans. Figure 5A displays the dose profile starting 1 cm inside the PTV and moving anteriorly-posteriorly toward the center of the spinal cord, thus displaying how the dose falls off as a function of distance from the PTV. Between 2,500 and 1,500cGy isodose lines, TrueBeam plans display a sharper dose gradient than the Halcyon™ plans, which is consistent with reported gradient measures in Figure 3A.

The dose fall-off for the treatment modalities can be seen in the axial slices, an example is shown in Figure 5B for case 2. The area of the low dose, as defined by the 1,500 cGy isodose line, increases between Halcyon™ 1.0, Halcyon™ 2.0, Millennium MLC, and HD MLC. The improvement in plan quality in Millennium MLC compared to Halcyon™ 2.0 is further verified by a decrease in the MCS of Millennium MLC as compared to Halcyon™ 2.0. Despite the ability to use both distal and proximal MLC layers to modulate, the MCS implies that the Halcyon™ 2.0 is not fully capable of matching the physical 0.5 cm of TrueBeam MLCs despite the theoretical modulation resolution of 0.5 cm.

### Limitations of the Study

This study investigates treatment plan quality and delivery accuracy for Halcyon™ and TrueBeam treatment modalities using gamma analysis, and assumes the machine meets standard mechanical and dosimetric specifications required by TG-142. However, this study does not address imaging requirements, patient setup reproducibility, and intra-fraction treatment monitoring.

Daily image guidance is essential to ensuring the steep dose gradient is positioned correctly with respect to the target volume and spinal cord positions. For image guidance, recommendations of Winston-Lutz test or daily MPC, which is required for Halcyon™ units, should always be performed prior to patient treatment. The Halcyon™ treatment platform requires daily imaging prior to patient treatment and includes the imaging dose within the treatment planning algorithm and optimization. For Halcyon™ 1.0, portal and MVCBCT using the primary 6FFF beam are available for image guidance. For Halcyon™ 2.0, portal and kV-CBCT is now available for image alignment. Detailed characterization of the Halcyon™ 2.0 system's kV CBCT performance has been reported by Cai et al. (14). Their results indicated that the Halcyon 2.0's CBCT provides larger field of view compared to Truebeam platform: ~50 cm in the axial plane, and 25 cm in the superior-inferior direction; with faster acquisition and improved noise performance when using iterative reconstruction. Based on their result, the on-board kV CBCT system for Halcyon 2.0 should be acceptable for spine SBRT localization.

Intra-fraction motion is of significant concern for spine SBRT since a patient's position must be maintained between planning and delivery to verify that the dose to the spinal cord is accurate. Rigid immobilization devices should be used to aid in patient setup reproducibility and minimize spinal motion. Since patients can be in substantial pain, any reduction in treatment time is valuable to assist in safe treatment delivery.

Current Halcyon™ implementation does not allow couch rotational correction, which is standard on TrueBeam linacs. This means if patient position needs to be adjusted rotationally (yaw correction), therapists would have to enter the treatment room to re-position the patient and re-image to confirm. This is a major limitation with the current Halcyon™ system design.

## Conclusion

The results in this study indicated Halcyon™ platform is capable of generating treatment plans that meet clinically accepted constraints and pass routine patient-specific quality assurance for delivery accuracy verification. For clinics that only have Halcyon™ as the sole treatment delivery option, administering spine SBRT is feasible and safe. However, caution should be taken on rigorous IGRT and patient repositioning, as the current system cannot provide automatic yaw correction for patient positioning. For clinics that have access to TrueBeam platforms, our data supports that for spine SBRT the Truebeam platform, especially when equipped with HD MLC, is still preferred over Halcyon™ both for superior cord sparing and automatic rotational correction capabilities for IGRT. Treatment time for both Halcyon™ and TrueBeam are comparable, as the former is currently limited by 800 MU/min dose rate. Future development on Halcyon™ to incorporate higher dose rate and 6-degrees-of-freedom couch will likely make it a more attractive option for spine SBRTs.

## Author Contributions

HP, IM, and TL assisted with treatment planning, plan quality analysis, and/or treatment plan delivery. AB provided classification scores by Cox et al. and Bilsky scores for this cohort. AG and JJ reviewed all treatment plans to determine if they met clinical standards and provided clinical guidance for the study. CW determined modulation complexity scores. WZ assisted with the extraction of control points for dual layer MLC by Halcyon. B-KT reviewed paper for both content and analysis providing valuable feedback for this work. LD and JMM help supervise and provided resources to perform the study. TL supervised all aspects of this work.

### Conflict of Interest Statement

This work is partially supported by Varian Medical Systems in that some of the data was generated by an evaluation system provided by the vendor. Varian Medical System had no influence in study design, data collection and analysis, decision to publish, or preparation of the manuscript. The brand name Halcyon™ is used with permission. JMM is on the advisory board of and obtained personal fee and grant funding unrelated to this work from Varian Medical Systems. LD received grant funding unrelated to this work from Varian Medical Systems. TL received personal fee unrelated to this work from Varian Medical Systems. The remaining authors declare that the research was conducted in the absence of any commercial or financial relationships that could be construed as a potential conflict of interest.
